# Current Strategies and Future Directions to Optimize ACL Reconstruction in Adolescent Patients

**DOI:** 10.3389/fsurg.2018.00036

**Published:** 2018-04-30

**Authors:** Dustin Jon Richter, Roger Lyon, Scott Van Valin, Xue-Cheng Liu

**Affiliations:** ^1^Department of Orthopaedic Surgery, Medical College of Wisconsin, Milwaukee, WI, United States; ^2^Department of Orthopaedic Surgery, Children’s Hospital of Wisconsin, Milwaukee, WI, United States

**Keywords:** anterior cruciate ligament, injury, reconstruction, adolescents, modeling

## Abstract

The incidence of anterior cruciate ligament (ACL) injuries in the pediatric population has risen in recent years. These injuries have historically presented a management dilemma in skeletally immature patients with open physes and significant growth remaining at time of injury. While those nearing skeletal maturity may be treated with traditional, transphyseal adult techniques, these same procedures risk iatrogenic damage to the growth plates and resultant growth disturbances in younger patients with open physes. Moreover, conservative management is non-optimal as significant instabilities of the knee remain. Despite the development of physeal-sparing reconstructive techniques for younger patients, there remains debate over which procedure may be most suitable on a patient to patient basis. Meanwhile, the drivers behind clinical and functional outcomes following ACL reconstruction remain poorly understood. Therefore, current strategies are not yet capable of optimizing surgical ACL reconstruction on an individualized basis with absolute confidence. Instead, aims to improve surgical treatment of ACL tears in skeletally immature patients will rely on additional approaches in the near future. Namely, finite element models have emerged as a tool to model complex knee joint biomechanics. The inclusion of several individualized variables such as bone age, three dimensional geometries around the knee joint, tunnel positioning, and graft tension collectively present a possible means of better understanding and even predicting how to enhance surgical decision-making. Such a tool would serve surgeons in optimizing ACL reconstruction in the skeletally immature individuals, in order to improve clinical outcomes as well as reduce the rate of post-operative complications.

## Introduction

Historically, anterior cruciate ligament (ACL) injuries were thought to be rare in the pediatric population. Instead, tibial eminence avulsion fractures were thought to be more common, as a result of the characteristically non-ossified bone found in adolescents (1). Recent studies however, indicate the incidence of ACL tears in the pediatric population has increased over the last 20 years, with the highest incidence occurring during high school years (2). Increased rates of ACL injuries are linked to both improved diagnostic techniques, as well as increased awareness within the medical community (3).

In the past, skeletally immature patients with ACL tears faced a management dilemma (4). In particular, the physes of the knee are of greatest anatomic concern due to the theoretical risk of physeal damage and growth disturbance. The distal femoral and proximal tibial physes contribute to 37 and 25% of overall limb length, respectively (5). As a result, pubertal status is of the utmost concern when evaluating surgical candidates’ risk of physeal disruption. In particular, sex differences between the age of typical skeletal maturity must be considered. In general, it is well-recognized females reach complete puberty including growth spurt and closure of the physes before their male counterparts. Skeletal maturity is reached at approximately age 14 years in girls, and age 16 in boys. Importantly, however, the amount of growth around the knee that remains after 12–13 years in girls and 14 years in boys is negligible (6). Therefore, until these ages are reached, surgeons must be aware of the growing physes when considering conservative versus operative management, and ultimately which ACL reconstruction strategy to utilize. Meanwhile, it must be kept in mind that each individual does not adhere to these guidelines, which means each case must be carefully analyzed in the setting of the particular patient and their growth status.

As previously mentioned, operative management restores stability of the knee but risks iatrogenic damage to the physis, which may ultimately lead to leg-length discrepancies and angular deformities of the lower extremity (7). Conversely, conservative management consisting of activity modification, rehabilitation exercises, and bracing avoids disruption of the physis, but has been associated with residual instability of the knee (8). These prolonged instabilities have been shown to lead to further complications and injuries, primarily meniscal tears and chondral degeneration (4). Therefore, the current consensus treatment for ACL tears in pediatric patients involves early surgical intervention restoring stability of the knee, which has now been associated with superior outcomes in comparison to conservative management (9).

However, ideal surgical techniques remain controversial due to insufficient data correlating these various techniques to clinical outcomes while taking into account the timing of surgery and rehabilitation regimes (8). The two primary surgical procedures in contention include transphyseal and physeal-sparing techniques. Anatomic, transphyseal reconstruction involves drilling tunnels though both the tibial and femoral physes, and is generally regarded with caution due to the risk of physeal disruption and growth disturbance. Physeal-sparing techniques, however, avoid drilling directly through the physis with modified all-epiphseal tunnels.

The tunnels used in transphyseal reconstructions account for little of the total cross sectional area of the physis. In addition, it has not been proven equivocally that drilling through the physis results in detectable valgus/varus deformities or growth disruption (2). Additionally, the question of which surgical procedure is optimal for each patient is complicated by variables such as the amount of remaining growth at time of surgery, and individual variations in geometry of the knee. Given advancements in finite element modeling capable of simulating biomechanical behavior of the knee joint (10), more sophisticated methods to optimize ACL reconstructions in skeletally immature patients on an individualized basis certainly seems an attainable goal in the relatively near future.

### Summary of Current Surgical Procedures

A summary of the following current surgical procedures is provided in Table 1. Previous studies have shown the ACL is composed of two main bundles consisting of the anteromedial (AM) and posterolateral (PL) constituents (15, 16). It has been determined the AM bundle assumes most of the strain in higher flexion angles, while the PL bundle takes up more strain in lower flexion angles (17). Together, the two bundles comprise the ACL, which prevents anterior tibial translation in addition to rotational stresses. Single bundle transtibial ACL reconstructions are often considered a traditional technique for restoring stability lost due to ACL rupture. While biomechanical studies have shown this technique can restore anterior-posterior stability, rotational kinematics are often not completely restored (18). Double bundle transtibial ACL reconstructions have been utilized to better restore stability and more normal knee kinematics (19). While this initially appears far more advantageous than the single bundle reconstructions, double bundle techniques are more technical procedures requiring longer operative times and more substantial bone loss. Overall, while some studies find double bundle reconstructions to provide better results, many other studies have failed to find significant differences in clinical outcomes (20). More recently, independent tunnel drilling for the femoral tunnel has been developed and accepted as a technique to more accurately replicate the anatomic femoral origin of the ACL (21).

**Table 1:** Summary of Surgical Procedures.

Surgical Procedure	Description	Advantages	Disadvantages
Anatomic Transtibial Single Bundle Reconstruction (11)	A femoral and tibial tunnel are drilled across the femoral and tibial physes, respectively, with a single-bundle graft which mostly reproduces the anteromedial (AM) bundle of the ACL.	Simple reconstruction which several studies have shown restore nearly normal knee kinematics.	Growth disturbances and angular deformities.
			Post-surgical stability and rotational laxity have been shown to be inferior to double bundle reconstructions.
Anatomic Transtibial Double Bundle Reconstruction (11)	Reconstructs AM and posterolateral (PL) bundles of anatomic ACL tendon separately.	The bundles are tensioned separately, resulting in even more natural tension patterns and restoring both anterior-posterior and rotational laxity.	Growth disturbances and angular deformities.
			Possible changes in knee joint kinematics may contribute to development of osteoarthritis (OA).
			Rupture of contralateral intact knee.
All epiphyseal technique of Lawrence et al. (12)	A lateral epiphyseal femoral tunnel and oblique epiphyseal tibial tunnel are drilled, avoiding the physes. The graft is secured in the tunnels via bioabsorbable screws.	Avoids disruption of open physes, decreasing chance of growth disturbances, leg length discrepancies and angular deformities.	The acute angle created by the tunnels may increase strain on the graft and increase risk of re-rupture.
			Growth disturbance by unidentified mechanism.
All epiphyseal technique of Janarv et al. (13)	A tibial epiphyseal tunnel is drilled, and the graft is placed posteriorly and over the femoral condyle in an “over-top” position on the femur and secured to the femoral metaphysis.	Avoids disruption of open physes, decreasing chance of growth disturbances, leg length discrepancies and angular deformities.	This non-anatomic reconstruction may not restore natural knee kinematics as well as anatomic reconstructions.
			Growth disturbance by unidentified mechanism.
Partial Physeal-Sparing Technique (14)	A transphyseal tibial tunnel disrupts the tibial physis, but the graft is fixed to the metaphysis of the lateral femur sparing the femoral physis.	Reduced risk of growth disruption, as femoral physis accounts for larger proportion of growth of lower limb.	Growth disturbance by disruption of tibial physis or unidentified mechanism.

As previously described, physeal-sparing techniques avoid disruption of the physes to decrease risk of growth abnormalities. In turn, this decreases the risk of leg length discrepancies and angular deformities. These procedures, however, are not without their disadvantages. Lawrence et al. describes an all-epiphyseal technique with a lateral femoral and oblique tibial tunnel, creating an acute angle the graft must traverse. Ultimately, this may lead to wear and unnecessary stress on the graft, increasing risk of re-rupture (12). To avoid this problem, an extraphyseal technique described by Janarv et al. places the graft in an “over-the-top” femoral position (13). While this technique avoids the graft being subjected to an acute angle, it is inherently non-anatomic and may not fully restore normal knee kinematics and stability.

Finally, partial physeal-sparing techniques (14) have been developed with the hopes of maintaining aspects of an anatomical reconstruction while minimizing risk of growth disturbance. In these procedures, a transphyseal tibial tunnel is drilled, but the femoral tunnel remains all-epiphyseal. As the femoral physis accounts for a larger proportion of limb growth, the avoidance of disrupting this physis is thought to minimize significant growth disturbances.

An additional procedure, which has just recently been developed, includes a hybrid physeal-sparing technique for ACL reconstruction. This technique provides an anatomic reconstruction that minimizes the risk of physeal damage but is also reproducible and less technical than the aforementioned physeal-sparing techniques. Willson et al. posits this technique provides high success rates with low morbidity in adolescents nearing skeletal maturity (22).

In sum, the variety of surgical procedures each provide a set of advantages and disadvantages which must be considered in pediatric patients with ACL ruptures. When choosing which procedure is optimal for a particular patient, factors including skeletal age and 3D geometry of the knee both affect surgical outcome. In regard to skeletal age, a current consensus has been well-described in the literature (23). In pre-pubescent patients with open physes and a great deal of anticipated growth remaining, physeal-sparing techniques are considered the most appropriate approach. To contrast, in adolescents with closed or closing physes, adult transphyseal techniques are regarded most highly due to the relatively low risk of iatrogenic damage and growth disturbances. However, many patients fall somewhere in the middle of this spectrum, and uncertainties often arise in skeletally-immature patients.

### Summary of Current Post Surgical Complications

As depicted in Table 2, a wide range of complications arises from both traditional transphyseal and physeal-sparing reconstructions. The main concern when performing ACL reconstructions has historically been disruption of the physes, leading to growth arrest. Ultimately, this disruption of growth leads to either leg length discrepancies or angular deformities of the leg (7). More recently, studies have suggested limb overgrowth leading to growth discrepancies may be underreported, and also pose a major clinical problem following ACL reconstruction in adolescents (30). Additional complications include re-rupture of the ACL graft, or even contralateral ACL tear. In fact, recent evidence suggests contralateral ACL tear may be more common than ACL graft rupture in patients following ACL reconstruction (31).

**Table 2:** Summary of Post-Surgical Complications.

Author/s	Surgical Procedure	Number of Subjects	Mean Age(Years)	Mean Follow-Up (Months)	Complications	Current Solution
Kumar et al. (24)	Transphyseal reconstruction	32	11.3	72.3	1 re-rupture	Repair rupture with additional ACL reconstruction
					1 valgus deformity	Surgical intervention to absolve angular deformity
Kocher et al. (25)	Transphyseal reconstruction	59	14.7	43	2 re-ruptures	Repair rupture with additional ACL reconstruction
Edwards and Grana (26)	Transphyseal reconstruction	20	13.7	34	2 re-ruptures	Repair rupture or lax graft with additional ACL reconstruction
					1 persistent laxity	
Chotel et al. (27)	Physeal-Sparing	2	8.5	24	2 limb overgrowths	Percutaneous epiphysiodesis
Shifflet et al. (28)	Physeal-Sparing	4	12.1	54	4 cases of growth arrest	Surgical intervention to correct limb length discrepancy
Kocher et al. (29)	Physeal-Sparing	44	10.3	44	2 re-ruptures	Repair rupture with additional ACL reconstruction
					4 repeat meniscal tears	Meniscal repair

In response to these complications, further surgical intervention or other procedures such as percutaneous epiphysiodesis are often required. Additional procedures not only result in additional financial burden for patients and their families, but also require additional recovery time and further delay until return to normal activities. Moreover, few if any prevention or predictive measures are currently in place to minimize the risk of these adverse outcomes. In addition, patients experience a wide range of functional outcomes affecting their daily lives and ability to reengage in sports or similar activities. These outcomes are assessed via common knee scores such as the International Knee Documentation (IKDC) subjective evaluation and Lysholm knee scores (32). Ideally, surgeons would strive for preventative strategies to minimize the risk of post-operative complications and to maximize clinical knee scores representing functional outcomes. However, the many mechanisms contributing to complications have either not been elucidated or are not clearly understood.

For example, in a systematic review by Collins et al., (33) physeal-sparing techniques counter-intuitively accounted for 25% of angular deformities and 47% of limb length discrepancy cases. This is unexpected, as it has long been thought sparing the physis decreases the risk of growth abnormalities. Furthermore, Collins et al. discovered limb overgrowth accounted for 62% of cases involving limb length discrepancy. Historically, growth arrest due to disruption of the physis has been the main concern after ACL reconstruction. Therefore, it suggests growth abnormalities are most likely under reported in the current literature, in addition to a gross lack of understanding underlying the precise causes and mechanisms leading to growth abnormalities following ACL reconstruction.

Meanwhile, it has been shown the 3D geometry of the distal femoral condyles has significant effects on knee joint kinematics (34). Therefore, it is likely individual differences in condylar shape and contour affect post-operative outcomes, in addition to the aforementioned skeletal age. As will next be discussed, current surgical procedures fail to address individual differences in condylar geometry during the procedure. Ultimately, this may lead to differences in tunnel orientations, angles, and positions between patients, which further contribute to post-operative complications and outcomes.

## Current Standard of Care Does Not Address Individual 3D Geometry of the Knee

Pena et al. have previously shown the angle of the femoral tunnel primarily affects tension of the graft, while the tibial tunnel significantly affects laxity and meniscal stresses (35). For example, tibial drill-guide angles between 55 and 65 degrees have been proposed as optimal angles for proper stress redistribution (36). Moreover, the graft tension is vitally important as enough tension is required to maintain stability, while a mechanism has been proposed where too much tension leads to physeal compression and ultimately damage (33). Furthermore, changes in tunnel positioning may take place in skeletally immature patients as growth around the knee potentially impacts reconstruction, which likely has direct impacts on *in vivo* graft tension as well. Of significance, a previous study has utilized multidector-row CT to demonstrate bone tunnel changes over time. Specifically, changes in angular geometry and actual movement of the articular outlets of tibial and femoral tunnels were measured at 1 year postoperatively compared to 3 weeks following the procedure (37). Therefore, it can be assumed tunnel position, angles, and draft tension cumulatively affect knee kinematics and functional outcomes.

Currently, a standard 7-mm-offset femoral tunnel guide is used to set the femoral guide pin in most conventional transphyseal ACL reconstructions. However, several studies such as Chung et al. (38) have suggested using a 10-mm-offset tunnel guide better enables the placement of a more anatomically positioned femoral tunnel which leads to greater stability, fewer complications, and better overall functional outcomes. This suggests very small differences in the positioning or placement of the guide pin have the potential to have tangible effects on clinical outcomes following ACL reconstructions. Meanwhile, it remains to be investigated how variation in individual distal femoral condyle 3D geometry and contour may affect positioning of the tunnel guide and guide pin. Based on Chung et al. suggesting even slight variation in the offset guide has important effects on tunnel positioning mimicking anatomical ACL placement, it is logical that small variations in distal femoral condyle geometry may affect tunnel angle and positioning as well. Although this remains to be investigated throughout the literature, anatomical variation in 3D geometry at the distal condyle likely effects tunnel placement, and in turn, has implications for vitally important clinical outcomes.

### Current Standard of Care Does Not Address Biomechanics of ACL Grafts

As previously illustrated, graft tension has important consequences on stability, functional outcomes, and complications such as graft rupture. However, the ideal graft tension which is determined during reconstruction remains a topic of contention within the surgical community. Most surgeons apply a “sufficient magnitude” of tension between 40 to 90 n to the graft at full extension (39). Kondo et al. illustrated how different tensioning strategies influenced functional outcomes such as knee flexion at 2 years of follow-up (40). Therefore, the great deal of variation in current graft tensioning protocols lends itself as a possible candidate to explain poorly understood differences in clinical outcomes. Additional factors compound the complexity of graft tension. For example, different graft types including patellar ligaments and hamstring tendons have been shown to exhibit different biomechanical properties such as strength and elasticity in long term follow-up studies (41).

Due to the ability of graft tension to affect functional outcomes, the need to better understand the many variables affecting tension after ACL reconstruction becomes imperative. Due to the fact that femoral condylar contour affects knee kinematics, it is plausible to conceive the 3D geometry has effects on long term graft tension. If these effects could be predicted through a computational model, graft tensioning protocols could be optimized to set initial graft tension more precisely to reduce post-surgical complications. Importantly, recent studies have validated the finite element modeling of such phenomena. Specifically, Pena et al. have demonstrated the plausibility of such modeling by using finite element analysis to study the effect of graft stiffness and graft tensioning on ACL reconstruction (42).

### Finite Element Models Capable of Simulating Biomechanical Behavior of Knee Joint

For decades, the biomechanics of the knee joint have been simulated by numerical means. Nonetheless, the intricate ability of the knee joint to provide both stability under high loads and simultaneous mobility continues to provide a challenging task to accurately model. Recently, modern non-linear, finite element models based on MRI have been shown to represent the knee with a “high degree of anatomical realism” (10). These models have since been developed to predict progressive aspects such degenerative pathology or surgical reconstruction. Most importantly, several studies have proven the ability of finite element analysis to model surgical repair and replacement strategies in relation to the knee (43–45). A review by Galbusera et al. highlights the progression and current utility of these models (10). Thus the simulation of the knee following surgical reconstruction is now achievable through the widespread validation of non-linear finite element packages and modern software with powerful capabilities to simulate the complex biomechanics of knee ligaments.

As previously illustrated, several factors pose the potential to be incorporated into finite element models to aid in the optimization of ACL reconstruction. For example, tunnel placement is of the foremost importance, but if and how it is affected by individual three-dimensional geometry of the knee would be a powerful modeling capability. Furthermore, skeletally immature patients pose the problem of growth around the knee taking place following reconstruction, which may resultantly alter tunnel positioning. Therefore, if bone age was to be incorporated into finite element models, the possibility of predicting tunnel position shifts could perhaps be anticipated to allow for optimal final tunnel location upon completion of growth around the knee. Finally, the many variables associated with graft tension such as tunnel angles, length, graft type, and tension set during the procedure, all have the potential to serve as valuable variables to better predict functional and clinical outcomes.

## Clinical Significance

The clinical significance of a predictive computational model capable of optimizing surgical outcomes are robust and far-reaching. Such an algorithm would not only allow surgeons to plan individualized procedures with unprecedented insight and ability, but would significantly increase functional outcomes while minimizing the risk of complications. With a greater understanding and ability to predict the effects of individual 3D geometry of the knee joint on tunnel positioning and graft tension, surgical techniques could be adjusted for individuals using pre-operative MRI data. Such a powerful tool may additionally be able to shorten surgical times based on pre-operative planning, reduce recovery time, and shorten rehabilitation regimens as patients experienced optimized reconstructions allowing for fast and complication-free recoveries.

The usefulness and validity of 3D-imaging of the knee was first established years ago by utilizing CT evaluation of tunnel placement (46). More recently, several studies have begun to highlight the utility of finite element modeling. For example, finite element analysis has been utilized to investigate the effects of tunnel orientation and stress distribution in ACL grafts (47), as well as to investigate the optimization of graft placement in ACL reconstruction (48). These studies provide early evidence of finite-element analysis aiding in the optimization of ACL reconstruction. The main limitation to these models is obviously the potential cost, which would potentially pose a barrier to widespread use on an individualized basis. Nonetheless, the development of select, cornerstone models in well-designed studies may be enough to glean useful information that can be applied to individual cases.

## Conclusions

Within the pediatric population, ACL injuries are becoming increasingly common. Current consensus suggests early reconstruction restores joint stability and is associated with superior long-term outcomes in comparison to solely conservative management. While physeal-sparing techniques have been developed for younger patients with significant growth remaining, these procedures have not eliminated the risks of post-operative complications and still pose distinct disadvantages. Therefore, in an effort to optimize ACL reconstruction in skeletally immature patients, the development and incorporation of finite element models poses a possible future direction. These models may potentially guide surgeons in choosing surgical techniques by including several individualized variables to allow optimal tunnel positioning, graft tension, and resulting biomechanics of the knee. Through these methods, the goal to continually strive to improve outcomes and reduce complications may be achieved.

## Ethics Statement

This study was reviewed and approved by the applicable IRB/ECs with respect to scientific content and compliance with applicable research and human subjects regulations.

## Author Contribution

All authors have met the criteria for authorship. They have made substantial contribution, have read and approved of the final submitted work, as well as agree to be accountable for all aspects of the work ensuring questions related to accuracy or integrity are appropriately investigated and resolved.

## Conflicts of Interest

The authors declare that the research was conducted in the absence of any commercial or financial relationships that could be construed as a potential conflict of interest.

## List of abbreviations

ACL: anterior cruciate ligament
AM: Anteromedial
PL: Posterolateral
